# Systematic review and meta-analysis of cognitive impairment in myalgic encephalomyelitis/chronic fatigue syndrome (ME/CFS)

**DOI:** 10.1038/s41598-021-04764-w

**Published:** 2022-02-09

**Authors:** Mehdi Aoun Sebaiti, Mathieu Hainselin, Yannick Gounden, Carmen Adella Sirbu, Slobodan Sekulic, Lorenzo Lorusso, Luis Nacul, François Jérôme Authier

**Affiliations:** 1 Neurology Department, Henri Mondor University Hospital, APHP, Créteil, France; 2 INSERM U955-Team Relaix, Faculty of Health, Paris Est-Creteil University, Créteil, France; 3 CRP-CPO, UR UPJV 7273, Université de Picardie Jules Verne, Amiens, France; 4 Central Military Emergency University Hospital, Titu Maiorescu University, Bucharest, Romania; 5 European Network On ME/CFS (EUROMENE), London, UK; 6 Department of Neurology, Faculty of Medicine Novi Sad, University of Novio Sad, Novio Sad, Serbia; 7 Neurology and Stroke Unit, Neuroscience Dept - ASST- Lecco, Merate Hospital, Merate, LC Italy; 8 Faculty of Infectious and Tropical Diseases, London School of Hygiene and Tropical Medicine, London, UK; 9 Neuromuscular Reference Center, Henri Mondor University Hospital, APHP, 51, Av. du Maréchal de Lattre de Tassigny, 94000 Créteil, France

**Keywords:** Neuroscience, Psychology, Neurology, Neurological disorders, Neuromuscular disease

## Abstract

Myalgic encephalomyelitis/chronic fatigue syndrome (ME/CFS) is commonly associated with cognitive complaints. To bring out the neuropsychological symptomatology inherent to ME/CFS, we conducted a systematic review according to PRISMA and MOOSE guidelines of the literature through the analysis of 764 studies published between 1988 and 2019 by using PubMed Central website and Clarivate analytics platform. We performed a meta-analysis to delineate an idea of the neuropsychological profile inherent in ME/CFS. The clinical picture typically affects visuo-spatial immediate memory (g = − 0.55, *p* = 0.007), reading speed (g = − 0.82, *p* = 0.0001) and graphics gesture (g = − 0.59, *p* = 0.0001). Analysis also revealed difficulties in several processes inherent in episodic verbal memory (storage, retrieval, recognition) and visual memory (recovery) and a low efficiency in attentional abilities. Executive functions seemed to be little or not affected and instrumental functions appeared constantly preserved. With regard to the complexity and heterogeneity of the cognitive phenotype, it turns out that determining a sound clinical picture of ME/CFS cognitive profile must go through a neuropsychological examination allowing a complete evaluation integrating the notion of agreement between the choice and the number of tests and the complexity intrinsic to the pathology.

## Introduction

Myalgic encephalomyelitis/chronic fatigue syndrome (ME/CFS) is a chronic disease defined by a severe and unexplained fatigue lasting for at least 6 months, involving central nervous system and immune system disorders, and resulting in a substantial reduction in occupational or leisure activities^1^. In healthy subjects, fatigue is usually proportional to effort or duration with a quick recovery and will recur to the same extent with the same effort or duration. In ME/CFS, the pathological threshold of fatigability is usually reached with minimal physical or mental exertion, with reduced ability to undertake the same activity within the same or several days. Other characteristic symptoms include self-reported memory and attention problems, tender lymph nodes, muscle pain, multi-joint pain, sore throat, headaches, un-refreshing sleep and post-exertional malaise. These clinical features are used for diagnostic criteria. Up to 20 case definitions have been proposed^2^ and those from Centers for Disease Control and Prevention (CDC)^3^ are the most commonly used^4^. The 2011 International Consensus Criteria^5^ are more detailed and therefore more complex to use in clinical practice. Compared to CDC criteria, they include three substantial changes: (1) The waiting period of 6 months before the diagnosis is no longer necessary; this is important because early diagnoses can help to better understand the early stages of the syndrome and quickly set up an appropriate care (which could reduce the severity and impact of disorders); (2) The post-exertional malaise is an necessary element in this classification; it is defined as an unusual intolerance to exercise with a recovery time higher than normal, characterized by: (a) a marked and rapid physical and/or cognitive fatigability at the least effort, (b) an exacerbation of symptoms after effort, (c) immediate or delayed post exercise exhaustion, (d) extended recovery period (24 h or more), (v) low threshold of physical and mental fatigability leading to substantial decrease in activity; (3) The identification of three groups of symptoms associated with chronic fatigue: (a) Indicators of an impairment of the nervous system: cognitive disorders, pain, sleep disturbances, sensory, perceptual and/or motor disturbances; (b) indicators of impairment of the immune, gastrointestinal and genitourinary systems: flu-like syndrome, increased susceptibility to infections, digestive disorders, urinary urgency, nocturia, hypersensitivity to food, drugs, odors or chemical substances; and (c) indicators of impairment of energy production/distribution systems: cardiovascular disorders, breathing difficulties, thermal deregulation, intolerance to extreme temperatures. These criteria allow precise case definition and fine stratification of patients. Recently, the Institute of Medicine (IOM)^6^ proposed to rename ME/CFS as systemic exertion intolerance disease (SEID) that describes the central elements of the disease. The report focuses on the adverse effect that physical, cognitive or emotional exertion can have on patients with this condition and acknowledges that this is a complex and severe disorder for which specific causes are not yet highlighted. For present study, we will retain the ME/CFS denomination, which is more frequently used. Cognitive manifestations are one of the most frequent and disabling symptoms associated with ME/CFS^7^, 89% of patients reporting memory and concentration problems^8^. From the 1980’s, cognitive complaints have led clinicians to perform formal neurocognitive assessment tests in ME/CFS patients. First results indicated that cognitive impairment mostly affects concentration and attention with a neuropsychological pattern suggesting an organic deficit, rather than cognitive dysfunction secondary to a mood disorder^9^. Subsequently, various studies provided additional evidences supporting that ME/CFS actually associates with cognitive deficits and, taken together, these researches allow to delineate a cognitive phenotype for ME/CFS patients^10–54^. Frequently observed features include ideomotor slowing down^11,12,15,17,21,30,40,42,44–47^, increased reaction time^12,13,15,24,31–37,39,41,54^ and attention deficit^18,31,40^. Regarding short-term memory, the visual modality was described as less efficient^29,33^, compared to the verbal one^15,21,24,52,54^. For long term memory, episodic memory impairment was commonly reported but its nature seems more difficult to determine. Indeed, the episodic memory processes encompasses three stages namely encoding, storage and retrieval^55^ to which are added the phases of recognition and consolidation of the memory trace^56^. While some studies reported the presence of deficit in consolidation (deferred recall deficit)^40^, others rightly raised uncertainties regarding the integrity of encoding phase^46^. Difficulties concerning executive functions have also been described, particularly concerning mental flexibility^39,40^, cognitive inhibition^51^ or the generation of information^29,31^. Finally, instrumental functions including calculation, language and visuo-construction, have been described as constantly efficient^10,12,14,15,17,21–23,30,31,33,44,46,49,52,54,57,58^. However, it must be noted that there is still some discrepancy between studies as some show deficits that others do not find. Moreover, some do not show any significant difference between the performances of ME/CFS patients and those of controls^57–62^. Finally, it seems not so easy to delineate a clear-cut neuropsychological phenotype of ME/CFS and, therefore, the cognitive symptoms can be regarded as heterogeneous and typically more variable than constant^63–67^.

This apparent heterogeneity of cognitive features described in ME/CFS could be linked, at least in part, to inter-study methodological divergences including the presence or absence of control groups, the inclusion criteria or the tests and standards used to quantify disorders^16^. On the other hand, it seems plausible to consider that the heterogeneous neuropsychological manifestations reflect the general heterogeneity of the syndrome in general. Indeed, chronic fatigue syndrome is a multiple entry entity^68^, for which symptomatology probable depends on the way and mode of entry. The heterogeneity of a cognitive profile is measured by the dissociations that will be observed between the cognitive domains tested. In fact, the wider is the screening, the more likely it is to perceive the presence of inter-functional performance differences. Although this principle adapts easily to individual evaluation, it is also suitable for group analysis and can detect both analogies and possible differences between patients presenting the same condition. In the end, there is to our knowledge no scientific work summarizing and analyzing the heterogeneous cognitive profiles of ME/CFS patients. In present the work, through the analysis of current literature by a systematic review and a meta-analysis approach, we attempted to delineate the cognitive profile of ME/CFS. From the obtained results, we finally propose a comprehensive neuropsychological battery applicable for the routine evaluation of ME/CFS patients.

## Material and methods

### Identification of studies

**Figure 1:**
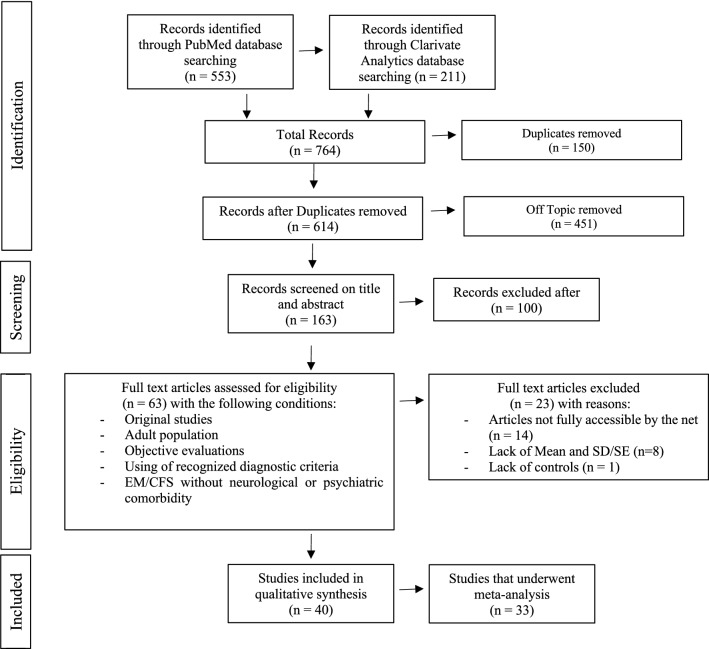
Flow chart depicting the study selection process through the phases of the systematic review and meta-analysis.

A systematic review was performed according to PRISMA (Preferred Reporting Items for Systematic Reviews and Meta-analyses)^69^ and MOOSE (Meta-analysis of Observational Studies in Epidemiology)^70^ guidelines (Fig. 1). We first conducted a literature review from PubMed Central website at the U.S. National Institutes of Health's National Library of Medicine (https://www.ncbi.nlm.nih.gov/pmc/). We used the keywords: “Myalgic encephalomyelitis/Chronic fatigue syndrome”, “Cognition”, and “Cognitive dysfunction”, and found 553 articles. With Clarivate analytics platform (Web of Science; https://clarivate.com) and the same key words, we obtained 211 articles, all of them having been retrieved through PubMed Central website (see supplementary material for PRISMA checklists).

We restricted the analysis to articles fulfilling the following inclusion criteria: (1) original study (reviews were rejected); (2) study conducted in an adult population (age > 16); (3) study using objective evaluations (self-reported measures were discarded); (4) ME/CFS diagnosed using the Center for Disease Control (CDC) criteria (Fukuda et al.^3^) or earlier variants; and (5) the inclusion of patients who did not have neurological or psychiatric pathology. From this sorting, we obtained 63 studies published between January 1988 and February 2019. Out of them, 14 could not be included because no full version was available. Finally, only the studies providing the means and the standard deviations/standard errors (SD/SE) and a comparison between ME/CFS patients and controls were included, which led to the exclusion of 9 other articles. A total of 40 studies have been analyzed. In these studies, ME/CFS patients’ neuropsychological evaluation scores were compared to those of matched controls (Fig. 1). The complete list of the 764 publications retrieved in PubMed Central website and Clarivate analytics platform is available on demand.

### Data preparation

Each test was categorized into one of nine cognitive domains, based on information provided in test corpus^71,72^. The cognitive domains were intellectual efficiency, reaction time, motor functioning, processing speed, attention, short term memory, long term memory, executive functions and instrumental functions (divided in three domains: language, calculation and visuo-construction).

All of the 40 eligible studies reported the data (Mean, SD/SE) necessary to calculate effect sizes for all tests that were used. Standard errors were converted into standard deviations. Some studies provided data for specific ME/CFS subgroups: gradual versus sudden onset of ME/CFS^22^; medicated versus medication free^39^ and morning versus afternoon test administration^31^. Due to the impossibility to examine the data for these too small subgroups, the means were therefore averaged (weighting by sample size) and the standard deviations combined^73^ to provide a single score for the ME/CFS group. In the case of studies integrating depressed and non-depressed ME/CFS patients, we focused on the results for ME/CFS non-depressed patients in order to fulfill exclusion criteria. No part of the study analyses was pre-registered prior to the research being conducted.

### Effect size calculations and analyses

Effect sizes were computed using Comprehensive Meta-Analysis Version 3^74^. Per outcome measure, the magnitude and direction of effect was calculated for each individual study. As we have disparities in terms of sample sizes small samples are associated with greater variability which affects the reliability of the effect size^75^, it is recommended to use weighted effect size. So, Hedges’ g was used to quantify effect sizes for each study and combined studies by using a fixed effects model. A fixed effects model was used because through our inclusions’ criteria, studies have all been conducted in the same population, they have used the same inclusion criteria and outcomes have been measured consistently. According to Cohen^76^, a small effect is defined as g = 0.2, a moderate effect as g = 0.5 and a large effect as g = 0.8. A medium effect size of 0.5 indicates that the mean test performance of the two groups differs by half a standard deviation. The effect sizes directions were determined automatically in function of the way of scoring for each test. For the weighted effect sizes 95% confidence intervals were additionally calculated^75^. Associated to the *P* value (a value of < 0.05 were considered significant) confidence intervals which do not span zero indicates a significant difference between the performance of the ME/CFS and healthy control groups. Importantly, only tests or scoring modality that were used by two or more studies were analyzed because effect sizes that are based on a single study do not provide a reliable measure of group differences^77^. This requirement led to exclude seven studies, resulting in a final total of 33 studies subjected to analysis (Fig. 1).

The selected inclusion criteria aimed to avoid as much bias as possible. However, this may lead to compare small numbers of studies, and thus to relativize the generalization of the outcomes of our analysis.

We used the Q statistic to measure effect sizes homogeneity. A significant Q index indicates that the variance of effect sizes in the population is greater than expected, compared to the sampling error. The percentage of variation across studies that is due to heterogeneity rather than chance (I2 statistic) was also calculated^78,79^. There is no heterogeneity when the I2 value is 0%. An I2 value of 25% indicates a low heterogeneity, 50% is moderate and 75% is high^79^. Results for which the level of heterogeneity is too high therefore cannot be considered and interpreted.

In case of heterogeneity, we tried to determine the origin by looking for a possible publication bias which is a common limitation of meta-analyses with the file-drawer effect^80^. A publication bias is defined by a trend to publish more studies showing statistically significant results than studies with nonsignificant results. This generates a type I publication bias error. Usually, the existence of a publication bias is characterized by an asymmetrical funnel shape, with a significant Egger’s test (*p* < 0.05). We thus used funnel plots and Egger’s regression tests to examine whether asymmetry due to publication bias was present in the study, and we also applied Rosenthal’s fail-safe N formula^80^ to estimate the number of unpublished studies with null findings.

### Data interpretation

The hypothesis that ME/CFS impacts cognitive functioning would be more reliable if first, the differences in performances between ME/CFS patients and healthy controls are at least moderate (g ≥ 0.5); and second, the fact that the effect size must be associated to a *P* value of < 0.05 and a 95% CI that does not span zero.

## Results

### Demographic data

The thirty-three studies included in the meta-analysis provided data for 1086 ME/CFS participants and 968 healthy controls (Table 1). Most participants were female (66%). Age (years; mean ± SD) was 39.5 ± 3.9 for ME/CFS patients, and 39.2 ± 4.4 for controls. ME/CFS and control patients were comparable in age (g = 0.016; *p* = 0.721) and displayed small difference for educational level (g = − 0.335; *p* < 0.05).

**Table 1:** Descriptive statistics for the study participants.

	ME/CFS				Healthy controls			
	N studies	N participants	Mean	SD	N studies	N participants	Mean	SD
Sample size	33	1086	33.62	23.2	33	968	29.33	19.44
**Gender**								
Female	28	719	25.68	20.25	28	637	22.75	16.6
Male	28	216	7.45	6.87	28	188	6.71	5.61
Age (years)	33	1086	39.47	3.92	33	968	39.21	4.43
Education^a^ (years)	21	637	14.51	1.11	21	536	15.1	1.16

*ME/CFS* myalgic encephalomyelitis/chronic fatigue syndrome, *N* number.

^a^Number of education years from age 6.

### Cognitive data

#### Intellectual efficiency

Intellectual efficiency is regarded as a sum of capacities that evolve over time, in line with the group of peers. These abilities are grouped into different cognitive subdomains, which are language skills, non-language reasoning skills (fluid reasoning), visuo-constructive skills, short-term memory and processing speed^81^. From the results obtained in these subdomains, one can establish the Intellectual Quotient (IQ). Twelve studies estimated the IQ of EM/CFS patients and controls. Some studies have mainly relied on the language domain: seven used the National Adult Test Reading; two based their analysis on the verbal comprehension index of the WAIS-R. Finally, three studies estimated IQ from a non-verbal indicator embodied by Raven’s progressive matrices (Table 2). All these tests producing small and no significant effect sizes (Table 2), indicating that people with ME/CFS did not differ from healthy controls on these measures.

**Table 2:** Intellectual efficiency: Hedge’s g effect sizes for each test, in descending order.

Test name	N studies	N CFS	N controls	Hedges'g	S.E	95% CI	*p* value	Q	*p*	I^2^ (%)	Study references
**Verbal**											
National adult reading test	7	210	164	− 0.07	0.11	− 0.272 to 0.140	0.533	7.35	0.29	18.40	Cockshell et al.^17^, Di Clementi et al.^57^, Fiedler et al.^24^, Joyce et al.^29^, Lawrie et al.^31^, Marshall et al.^35^, Robinson et al.^44^
WAIS-R Verbal	2	34	33	− 0.10	0.24	− 0.571 to 0.364	0.664	0.03	0.86	0.00	Claypoole et al.^15^, DeLuca et al.^20^
**Non-verbal**											
Standard progressive matrices	3	84	77	− 0.17	0.16	− 0.476 to 0.136	0.276	0.50	0.78	0.00	Michiels et al.^39,40,41^

*WAIS* Wechsler adult intelligence scale, *ME/CFS* myalgic encephalomyelitis/chronic fatigue syndrome, *N* number.

#### Reaction time

**Figure 2:**
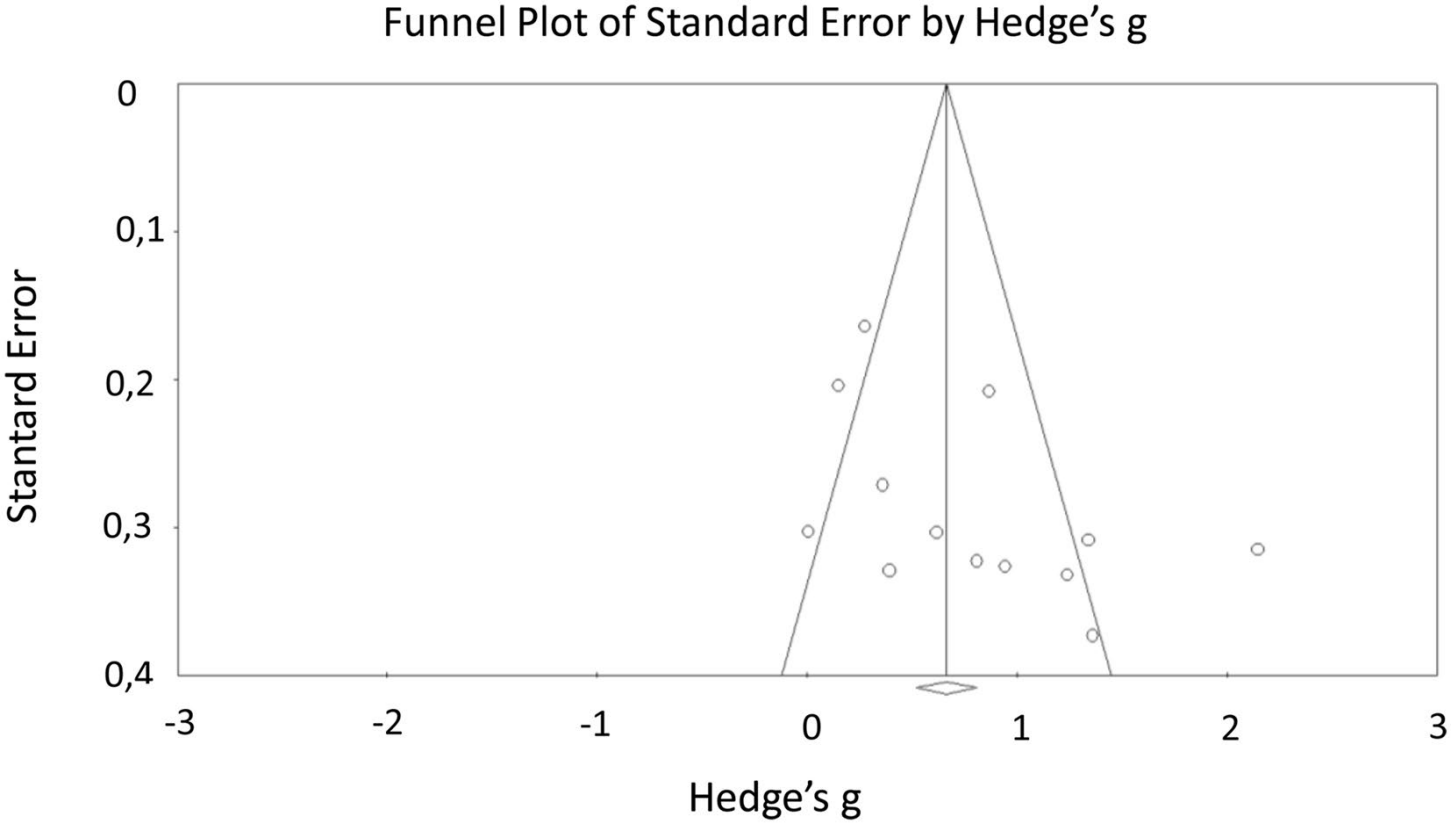
Funnel plot of standard error by Hedge's g for simple reaction time.

**Figure 3:**
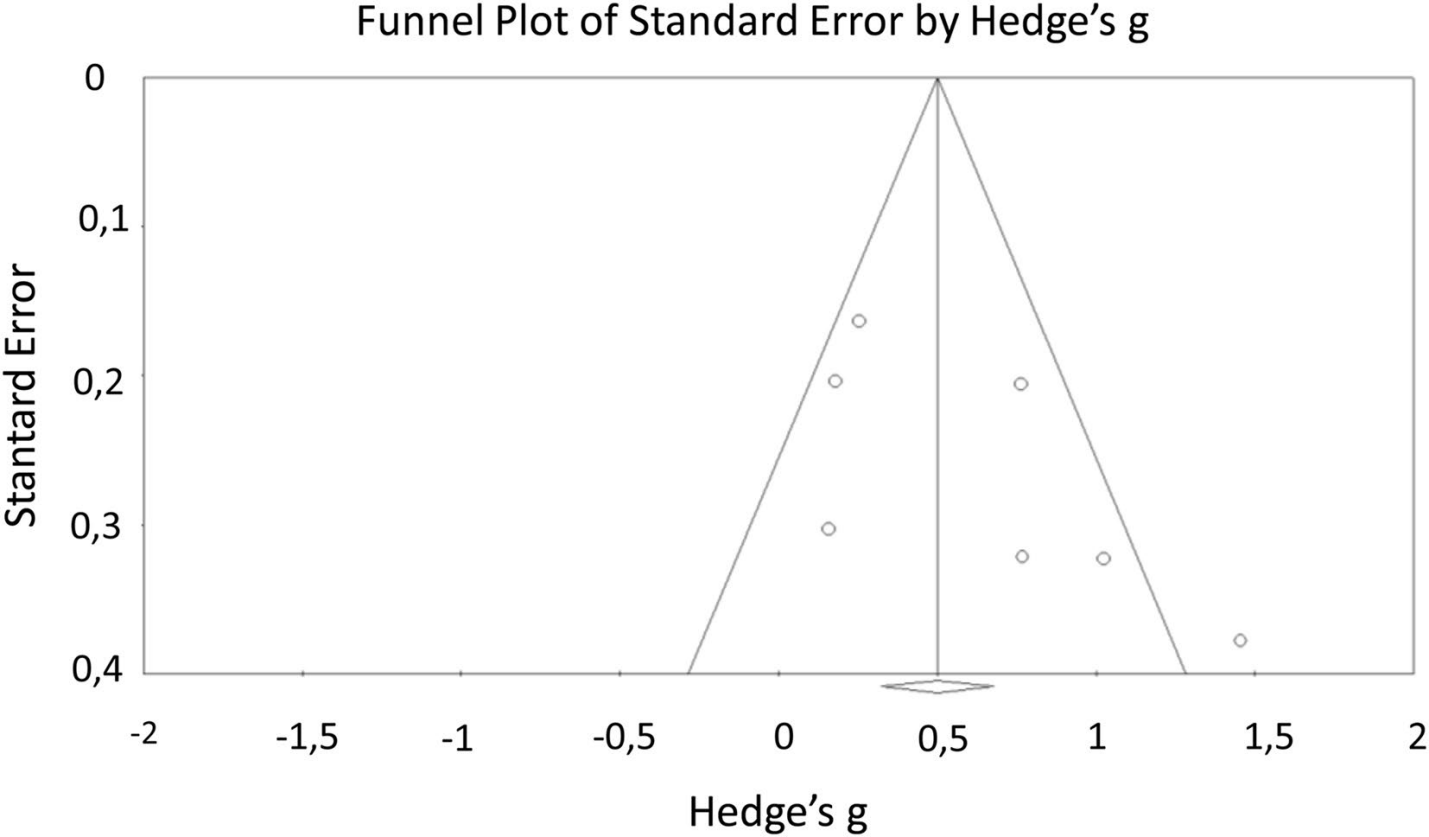
Funnel plot of standard error by Hedge's g for choice reaction time.

Reaction time is the time between stimulation and response, depending on the path taken by the nervous message and summing the perception of message, it is the integration, and the elaboration of a response. There are three types of reaction time: (1) simple reaction time between a unique stimulus and expected reaction; the response being expected, the motor response takes place before the appearance of the stimulus and "waits" for a trigger; (2) the reaction time of choice, or complex reaction time: time between two stimulus and the induced response; in this case, the preparation of the response takes place only after the appearance of the double stimulus ; (3) semi-complex reaction time: time between a stimulus requiring treatment and an expected response^82,83^. Thirteen studies compared the simple reaction time of ME/CFS patients to those of controls and seven analyzed the results relating to choice reaction time (see Table 3). There were significant moderate effect sizes for these two tests, indicating that the ME/CFS group showed an increased reaction time (Table 3). However, the significant high heterogeneity (Q = 54.09, *p* < 0.005; I^2^ = 77.81% for Simple reaction time test and Q = 17.36, *p* = 0.01; I^2^ = 65.43% for Choice reaction time test) between the studies did not allow us to conclude that there is a true group effect. Due to this heterogeneity, regarding Simple reaction time test, we generated a funnel plot and used Egger’s regression intercept in order to identify the possible presence of asymmetry due to publication bias. Visual inspection of the figure (see Fig. 2) showed a little asymmetry, but the Egger’s regression was not significant (*p* = 0.06). Rosenthal’s fail-safe N indicated that 309 additional studies with null results would be required to reveal a difference between CFS patients and controls. For Choice reaction time test, visual inspection of the figure (see Fig. 3) did not show a clear asymmetry, and the Egger’s regression was not significant (*p* = 0.10). Rosenthal’s fail-safe N indicated that 61 additional studies with null results would be required to reveal a difference between CFS patients and controls.

**Table 3:** Reaction time: Hedge’s g effect sizes for each test, in descending order.

Test name	N studies	N CFS	N controls	Hedges'g	S.E	95% CI	*p* value	Q	*p*	I^2^ (%)	Study references
Simple reaction time (ms)	13	24	403	0.66	0.07	0.520–0.808	0.0001	54.09	0.00	77.81	Cockshell et al.^17^, Capuron et al.^13^, Claypoole et al.^15^, Fiedler et al.^24^, Lawrie et al.^31^, Mahurin et al.^32^, Majer et al.^33^, Marcel et al.^34^, Marshall et al.^36^, Marshall et al.^35^, Michiels et al.^39,41^, Smith et al.^51^
Choice reaction time	7	239	282	0.50	0.09	0.323–0.678	0.0001	17.36	0.01	65.43	Cockshell et al.^17^, Capuron et al.^13^, Mahurin et al.^32^, Majer et al.^33^, Marshall et al.^36^, Marshall et al.^35^, Michiels et al.^39^

*ME/CFS* myalgic encephalomyelitis/chronic fatigue syndrome, *N* number.

#### Motor functioning

**Figure 4:**
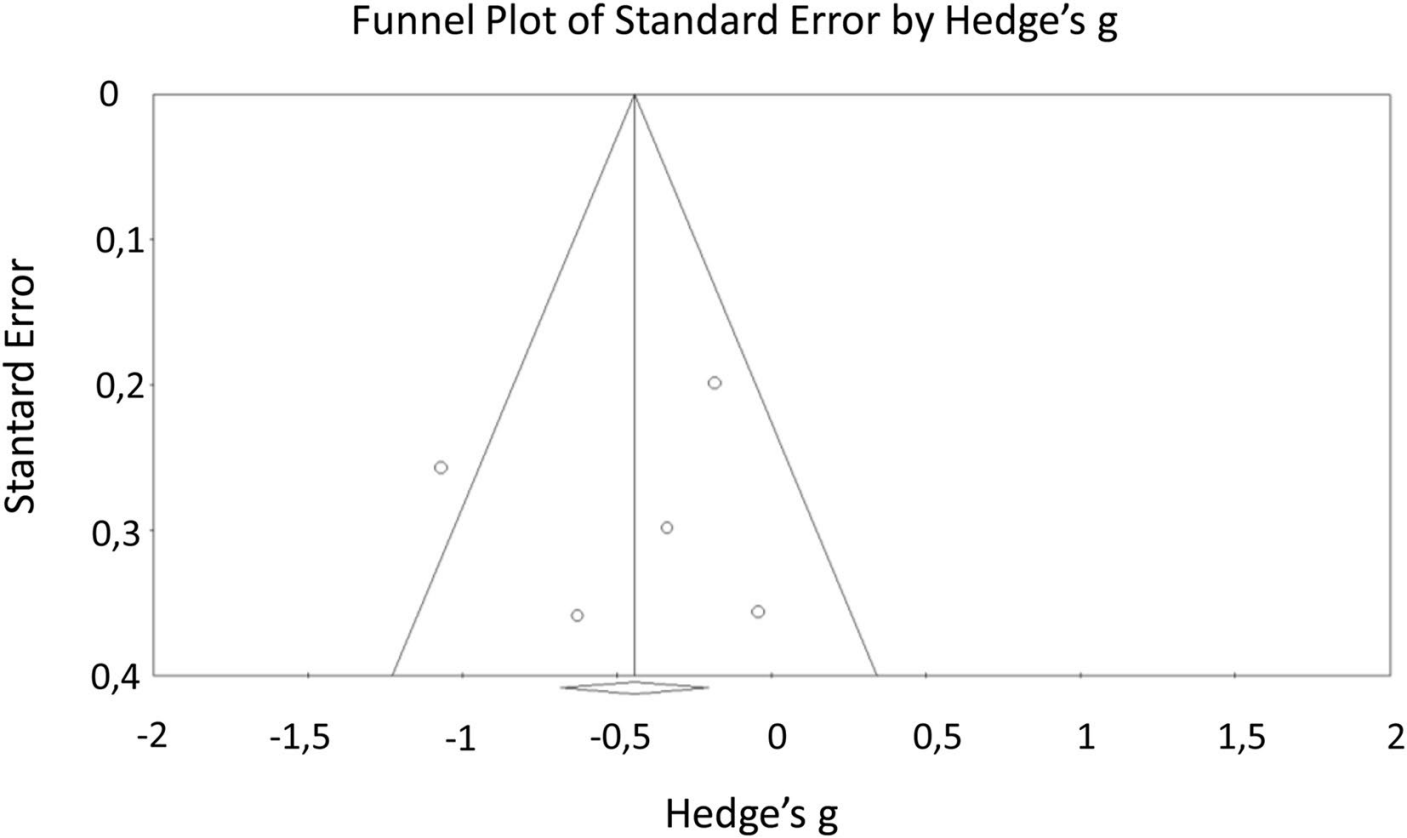
Funnel plot of standard error by Hedge's g for Finger tapping test.

This section groups together motor activity assessments, including reaction time (see supra) as well as the speed of movement associated with dexterity (Grooved Pegboard) and coordination (Finger Tapping Test). Seven studies assessed speed, dexterity and motor coordination and four evaluated the reaction time linked to movement (Table 4). There was a small to moderate effect size in terms of reaction time, coordination speed related to movement. No effect size was observed for the Grooved Pegboard Test (Table 4). Nevertheless, results related to the Finger Tapping Test could not be interpreted because of the significant moderate heterogeneity (Q = 9.29, *p* = 0.05; I^2^ = 56.96%) between studies. Due to this heterogeneity, we generated a funnel plot and used Egger’s regression intercept in order to identify the possible presence of asymmetry due to publication bias. Visual inspection of the figure (see Fig. 4) did not show a clear asymmetry, and the Egger’s regression was not significant (*p* = 0.87). Rosenthal’s fail-safe N indicated that 12 additional studies with null results would be required to reveal a difference between CFS patients and controls.

**Table 4:** Motor functioning: Hedge’s g effect sizes for each test, in descending order.

Test name	N studies	N CFS	N controls	Hedges'g	S.E	95% CI	*p* value	Q	*p*	I^2^ (%)	Study references
Choice movement time	2	101	157	0.45	0.13	0.196–0.701	0.001	0.46	0.50	0.00	Capuron et al.^13^, Majer et al.^33^
Finger tapping test	5	138	135	− 0.44	0.12	− 0.681 to − 0.204	0.0001	9.29	0.05	56.96	Cockshell et al.^17^, Claypoole et al.^15^, Michiels et al.^40^, Neu et al.^42,43^
Simple movement time	2	101	157	0.31	0.13	0.064–0.566	0.014	0.44	0.51	0.00	Capuron et al.^13^, Majer et al.^33^
Grooved perboard	2	40	40	0.05	0.22	− 0.383 to 0.478	0.828	0.54	0.46	0.00	Claypoole et al.^15^, Fiedler et al.^24^

*ME/CFS* myalgic encephalomyelitis/chronic fatigue syndrome, *N* number.

#### Processing speed

Ideo-motor speed refers to the time it takes a person to do a mental task. It is the speed with which a person understands and reacts to information, whether by visual means (letters and numbers), hearing (language) or movement. Processing speed is therefore the time we take between the moment we receive the stimulation and when we respond^84^. Fourteen studies proposed tests assessing ideo-motor processing speed. Seven tested the processing speed via symbol tests (WAIS and SDMT) with a strong graphic bias. Nine used TMT A with a minor graphic component. Finally, six studies assessed processing speed through the single visual input channel via the Stroop test (in different conditions that do not involve inhibition abilities) (Table 5).

**Table 5:** Processing speed: Hedge’s g effect sizes for each test, in descending order.

Test name	N studies	N CFS	N controls	Hedges'g	S.E	95% CI	*p* value	Q	*p*	I^2^ (%)	Study references
Symbol (WAIS)	4	117	116	− 0.70	0.13	− 0.960 to − 0.436	0.0001	2.63	0.45	0.00	Michiels et al.^40^, Neu et al.^42,43^, Vercoulen et al.^54^
Symbol digit modalities test	4	90	75	− 0.59	0.16	− 0.898 to − 0.276	0.0001	1.61	0.66	0.00	Claypoole et al.^15^, Fiedler et al.^24^, Krupp et al.^30^, Lawrie et al.^31^
**Stroop**											
Colors/words cotation 1	2	71	40	− 0.82	0.21	− 1.224 to − 0.412	0.0001	0.00	0.98	0.00	Marshall et al.^35^, Robinson et al.^44^
Colors/words cotation 2	2	58	58	− 0.76	0.19	− 1.134 to − 0.389	0.0001	0.76	0.38	0.00	Fiedler et al.^24^, Metzger et al.^59^
Colors	4	117	86	− 0.23	0.15	− 0.524 to 0.058	0.117	27.01	0.00	88.89	Claypoole et al.^15^, Marshall et al.^35^, Ray et al.^60^, Robinson et al.^44^
Words	4	117	86	− 0.23	0.15	− 0.520 to 0.062	0.123	26.17	0.00	88.54	Claypoole et al.^15^, Marshall et al.^35^, Ray et al^60^, Robinson et al.^44^
TMT A	9	285	225	0.40	0.09	0.222–0.578	0.0001	16.22	0.04	50.68	Claypoole et al.^15^, De Luca et al.^21^, Krupp et al.^30^, Lawrie et al.^31^, Michiels et al.^40^, Michiels et al.^39^, Robinson et al.^44^, Tiersky et al.^52^, Vercoulen et al.^54^

*WAIS* Wechsler adult intelligence scale, *TMT* trail making test, *ME/CFS* myalgic encephalomyelitis/chronic fatigue syndrome, *N* number.

Statistical analysis showed a moderate to large effect size with respect to processing speed, whether it involved a strong graphomotor act or a grueling reading speed (significant and large effect size mainly for the Color/Word condition in Stroop) (Table 5).

#### Attention

Attention refers to a cognitive enhancer allowing information to be processed consciously. This is a resource that can be allocated for a prolonged period to a target that could be chosen among many other stimuli (sustained and selective attention) or to several targets simultaneously (divided attention)^85^. Nineteen studies proposed tests assessing attention. Thirteen studies proposed an evaluation of attention through the auditory canal via the Paced Auditory Serial Addition Test (PASAT) under different conditions (Table 6) involving sustained attention skills but inducing executive bias through the involvement of working memory. Six studies used visual canal via the Continuous Performance Test (CPT) and the Rapid Visual information Processing (RVP) (Table 6) allowing the assessment of sustained and selective attention. There is a significant good effect size for PASAT (4 s), witnessing the impact of EM/CFS on sustained attention skills at auditory input. The results appeared less clear with visual input attention, for which there is only the CPT (Number no distraction condition) which showed a significant moderate effect size, in favor of an underperformance in selective and sustained attentional capacities at visual entry in ME/CFS patients compared to controls (Table 6).

**Table 6:** Attention: Hedge’s g effect sizes for each test, in descending order.

Test name	N studies	N CFS	N controls	Hedges'g	S.E	95% CI	*p* value	Q	*p*	I^2^ (%)	Study references
**Auditive input**											
Paced auditory serial addition test (4 s)	3	84	62	− 0.75	0.17	− 1.086 to − 0.409	0.0001	2.35	0.31	15.07	Constant et al.^18^, Lawrie et al.^31^, Michiels et al.^41^
Paced auditory serial addition test (Total corrects)	9	261	239	− 0.46	0.09	− 0.634 to − 0.282	0.0001	4.89	0.77	0.00	Cockshell et al.^17^, Cockshell et al. (2014), Claypoole et al.^15^, DeLuca et al.^20^, DeLuca et al.^21^, DeLuca et al.^22^, Marshall et al.^35^, Schmalin et al.^61^, Tiersky et al.^52^
Paced auditory serial addition test (2 s)	2	53	38	− 0.37	0.22	− 0.788 to 0.052	0.086	1.04	0.31	3.92	Lawrie et al.^31^, Short et al.^49^
**Visual input**											
Continuous performance test (number no distraction)	2	47	32	− 0.50	0.23	− 0.957 to − 0.046	0.031	0.01	0.94	0.00	Marshall et al.^35,36^
Continuous performance test (number distraction)	2	47	32	− 0.48	0.23	− 0.933 to − 0.022	0.04	0.56	0.46	0.00	Marshall et al.^35,36^
Continuous performance test (shapes distraction)	2	47	32	− 0.45	0.23	− 0.906 to 0.007	0.05	2.13	0.14	53.01	Marshall et al.^35,36^
Rapid visual information processing (A')	2	101	157	− 0.28	0.13	− 0.531 to − 0.030	0.028	0.00	0.99	0.00	Capuron et al.^13^, Majer et al.^33^
Continuous performance test [reaction time (ms)]	2	47	43	0.26	0.21	− 0.145 to 0.672	0.206	0.00	1.00	0.00	Fiedler et al.^24^, Marcel et al.^34^
Continuous performance test (shapes no distraction)	2	47	32	− 0.21	0.23	− 0.662 to 0.241	0.361	1.03	0.31	2.60	Marshall et al.^35,36^
**Other**											
Mental contol	2	49	42	− 0.40	0.21	− 0.813 to 0.006	0.05	0.07	0.79	0.00	Grafman et al.^25^, Marcel et al.^34^

*ME/CFS* myalgic encephalomyelitis/chronic fatigue syndrome, *N* number.

However, the fact that the effect size is at least moderate in only one of the tests or subtests identified in each entry canal leads to relativize the idea of a clear or constant impairment of attentional capacities in ME/CFS patients.

#### Short term memory

Short-term memory refers to immediate (retention in memory by loop repetition system and re-use as given of the information) and working (manipulation of the information kept in memory) memory^84^. Fourteen studies proposed tests evaluating short-term memory abilities in both modalities, eleven in verbal one and four in visuo-spatial domain (Table 7).

**Table 7:** Short-term memory: Hedge’s g effect sizes for each test, in descending order.

Test name	N studies	N CFS	N controls	Hedges'g	S.E	95% CI	*p* value	Q	*p*	I^2^ (%)	Study references
**Visual modality**											
Spatial span forward	2	71	40	− 0.55	0.20	− 0.952 to − 0.151	0.007	2.03	0.15	50.64	Joyce et al.^29^, Robinson et al.^44^
Spatial working memory (between search errors)	2	101	157	0.26	0.13	0.012–0.514	0.04	0.00	0.94	0.00	Capuron et al.^13^, Majer et al.^33^
Spatial working memory (strategy score)	2	101	157	0.36	0.13	0.105–0.609	0.006	3.28	0.07	69.50	Capuron et al.^13^, Majer et al.^33^
**Verbal modality**											
Digit span backward	8	251	195	− 0.37	0.10	− 0.566 to − 0.182	0.0001	24.50	0.00	71.43	De Luca et al.^22^, Fiedler et al.^24^, Lawrie et al.^31^, Michiels et al.^39^, Neu et al.^42^, Robinson et al.^44^, Tiersky et al.^52^, Vercoulen et al.^54^
Digit span forward	11	359	261	− 0.23	0.08	− 0.387 to − 0.064	0.006	7.64	0.66	0.00	Claypoole et al.^15^, De Luca et al.^22^, Fiedler et al.^24^, Lawrie et al.^31^, Marcel et al.^34^, Michiels et al.^39^, Neu et al.^42^, Robinson et al.^44^, Smith et al.^51^, Tiersky et al.^52^, Vercoulen et al.^54^

*ME/CFS* myalgic encephalomyelitis/chronic fatigue syndrome, *N* number.

In verbal modality, the studies used classical tests of span in forward and backward conditions which allowed for a complete analysis in short-term memory. The results showed a significant effect size but too small to assess a deficit in this modality in ME/CFS patients (Table 7). For visuo-spatial modality, only two tests were used: (1) the Spatial Span Forward, which allows specific evaluation of immediate memory in visual modality; and (2) Spatial Working Memory which requires retention and manipulation of visuospatial information. Spatial Working Memory also involves executive functions and provides a measure of strategy as well as working memory errors.

The results showed a moderate and significant effect size with Spatial Span. There is only a small effect size for the Spatial Working Memory test, for both conditions (Table 7).

This indicates the tendency of ME/CFS patients to underperform in immediate memory in visuospatial modality. However, the non-significant but moderate heterogeneity (Q = 2.03, *p* = 0.15; I^2^ = 50.64%) invites us to consider the results with caution, although results are not invalidated. On the other hand, the executive component inherent in short-term memory (working memory) would seem relatively equivalent between ME/CFS and controls.

There is just a small effect size for verbal modality in both condition (forward and backward) which suggests that ME/CFS patients perform in a relatively equivalent way to controls in this domain.

#### Long term memory

Evaluation of long term memory corresponds to episodic memory, which can be defined as a multi-step process including the following different phases: encoding, storage/consolidation, recovery, and recognition of information recorded in a precise spatial and temporal context^55,86^. This mechanism is underpinned by a plural physiological mechanism including the Papez circuit^87–90^.

Twenty studies included tests assessing episodic memory, fifteen in verbal modality and twelve in visual/visuospatial modality (Table 8).

**Table 8:** Long term memory: Hedge’s g effect sizes for each test, in descending order.

Test name	N studies	N CFS	N controls	Hedges'g	S.E	95% CI	*p* value	Q	*p*	I^2^ (%)	Study references
**Episodic**											
*Verbal modality*											
California verbal learning test T score List A (Trials 1–5)	7	231	214	− 0.50	0.10	− 0.691 to − 0.315	0.0001	11.29	0.08	46.86	Cockshell et al.^17^, De Luca et al.^21,22^, Fiedler et al.^24^, Michiels et al.^39^, Tiersky et al.^52^, Vercoulen et al.^54^
California verbal learning test long delay free recall	7	263	246	− 0.36	0.09	− 0.534 to − 0.184	0.0001	9.59	0.14	37.46	Cockshell et al.^17^, Cockshell et al. (2014), De Luca et al.^21,22^, Michiels et al.^39^, Tiersky et al.^52^, Vercoulen et al.^54^
California verbal learning test short delay free recall	7	263	246	− 0.35	0.09	− 0.527 to − 0.179	0.0001	5.73	0.45	0.00	Cockshell et al.^17^, Cockshell et al. (2014), De Luca et al.^21,22^, Michiels et al.^39^, Tiersky et al.^52^, Vercoulen et al.^54^
California verbal learning test long delay cued recall	4	177	173	− 0.27	0.11	− 0.483 to − 0.064	0.01	2.40	0.49	0.00	Cockshell et al.^17^, Cockshell et al. (2014), De Luca et al.^21^, Vercoulen et al.^54^
California verbal learning test recognition	5	197	195	− 0.26	0.10	− 0.452 to − 0.058	0.011	1.80	0.77	0.00	Cockshell et al.^17^, Cockshell et al. (2014), De Luca et al.^21^, Michiels et al.^39^, Vercoulen et al.^54^
California verbal learning test short delay cued recall	4	177	173	− 0.21	0.11	− 0.422 to − 0.004	0.045	3.37	0.34	11.11	Cockshell et al.^17^, Cockshell et al. (2014), De Luca et al.^21^, Vercoulen et al.^54^
Weschler logical memory 2	4	98	79	− 0.43	0.15	− 0.729 to − 0.137	0.004	1.15	0.77	0.00	Claypoole et al.^15^, De Luca et al.^21^, Grafman et al.^25^, Tiersky et al.^52^
Weschler logical memory 1	6	141	122	− 0.41	0.12	− 0.651 to − 0.166	0.001	5.98	0.31	16.43	Claypoole et al.^15^, De Luca et al.^21^, Grafman et al.^25^, Krupp et al.^30^, Short et al.^49^, Tiersky et al.^52^
Weschler verba paired associates 2	2	42	39	− 0.29	0.22	− 0.727 to 0.138	0.182	1.74	0.19	42.67	Claypoole et al.^15^, Grafman et al.^25^
Weschler verba paired associates 1	2	42	39	0.10	0.22	− 0.327 to 0.53	0.642	0.24	0.62	0.00	Claypoole et al.^15^, Grafman et al.^25^
*Buschke selective reminding test*											
Long term retrieval	2	64	55	− 0.64	0.19	− 1.005 to − 0.274	0.001	0.05	0.82	0.00	Michiels et al.^40,41^
Delayed recall	3	75	73	− 0.58	0.17	− 0.908 to − 0.259	0.0001	1.13	0.57	0.00	Krupp et al.^30^, Marshall et al.^35^, Michiels et al.^40^
Long term storage	3	84	75	− 0.57	0.16	− 0.883 to − 0.256	0.0001	0.07	0.96	0.00	Marshall et al.^35^, Michiels et al.^40,41^
Delayed recognition	2	64	55	− 0.18	0.24	− 0.655 to 0.288	0.45	0.00	1.00	0.00	Michiels et al.^40,41^
*Rey auditory verbal learning test*											
Total 8	2	52	37	− 0.80	0.22	− 1.233 to − 0.360	0.0001	0.63	0.43	0.00	Claypoole et al.^15^, Lawrie et al.^31^
Total 7	4	118	73	− 0.68	0.15	− 0.978 to − 0.373	0.0001	2.51	0.47	0.00	Claypoole et al.^15^, Lawrie et al.^31^, Neu et al.^42^, Robinson et al.^44^
Recognition	3	96	51	− 0.49	0.18	− 0.832 to − 0.139	0.006	2.79	0.25	28.30	Lawrie et al.^31^, Neu et al.^42^, Robinson et al.^44^
Total 6	3	88	58	− 0.41	0.17	− 0.754 to − 0.075	0.017	2.10	0.35	4.96	Claypoole et al.^15^, Neu et al.^42^, Robinson et al.^44^
Total 1–5	4	118	73	− 0.41	0.15	− 0.707 to − 0.113	0.007	0.99	0.80	0.00	Claypoole et al.^15^, Lawrie et al.^31^, Neu et al.^42^, Robinson et al.^44^
*Visual modality*											
Rey Osterrieth complex figure recall 3 min (Cotation b)	3	92	71	− 0.67	0.16	− 0.979 to − 0.351	0.0001	0.17	0.92	0.00	De Luca et al.^21,22^, Tiersky et al.^52^
Rey Osterrieth complex figure recall 20 min (Cotation b)	2	62	51	− 0.64	0.19	− 1.013 to − 0.263	0.001	0.10	0.75	0.00	De Luca et al.^21,22^
Rey Osterrieth complex figure recall 3 min (Cotation a)	5	196	198	− 0.22	0.10	− 0.421 to − 0.027	0.026	4.28	0.37	6.56	Cockshell et al.^17^, Cockshell et al. (2014), Claypoole et al.^15^, Short et al.^49^, Vercoulen et al.^54^
Rey Osterrieth complex figure recall 20 min (Cotation a)	3	122	122	− 0.06	0.13	− 0.313 to 0.185	0.616	1.77	0.41	0.00	Cockshell et al.^17^, Cockshell et al. (2014), Claypoole et al.^15^
Spatial recogniton memory (%)	2	101	157	− 0.32	0.13	− 0.568 to − 0.066	0.013	0.46	0.50	0.00	Capuron et al.^13^, Majer et al.^33^
Pattern recognition memory (%)	2	101	157	− 0.04	0.13	− 0.286 to 0.214	0.776	1.55	0.21	35.58	Capuron et al.^13^, Majer et al.^33^
Visual reproduction 1	2	38	35	− 0.57	0.23	− 1.028 to − 0.107	0.016	0.97	0.32	0.00	Fiedler et al.^24^, Grafman et al.^25^
Visual reproduction 2	2	38	35	− 0.47	0.23	− 0.924 to − 0.008	0.046	1.38	0.24	27.52	Fiedler et al.^24^, Grafman et al.^25^

*ME/CFS* myalgic encephalomyelitis/chronic fatigue syndrome, *N* number.

For verbal episodic memory, studied papers uses California Verbal Learning Test (CVLT; n = 7), Buschke Selective Reminding Test (SLT; n = 4), Rey Auditory Verbal Learning Test (RAVLT; n = 4), Weschler Logical Memory (n = 6) and Verbal Paired Associates (n = 2). Analysis revealed a significant effect size between moderate and large for several subtests evaluating the ability to strategically search for information in episodic memory (; long term retrieval and delayed recall in SLT; delayed recall in RAVLT), which is the executive component of episodic memory. We also observed a significant and moderate effect size for the storage (long term storage in SLT) and recognition (recognition phase in RAVLT) phases (Table 8).

These elements suggested that ME/CFS patients are less efficient than controls for each process inherent in episodic memory.

For visual episodic memory, the tests that were used, evaluated two processes inherent to episodic memory, namely the capacities of recovery (recall in Rey-Osterrieth Complex Figure [ROCF] and Visual Reproduction Test) and the recognition capacities (Spatial Recognition Memory and Pattern Recognition Memory) (Table 8).

Statistical analysis revealed significant moderate to large effect sizes for the recall phases (ROCF 3 min-recall and Visual reproduction 1) of information stored in episodic memory (Table 8).

#### Executive functions

Executive functions refer to functions of control and regulation of cognitive and behavioral activity^91^. These functions are anatomically underpinned by fronto-subcortical loops and related networks^91–93^. Twenty-one studies evaluated executive functions. The tests used in analyzed studies made possible to check the performances of ME/CFS patients in several executive functions, namely planning capacities (Stockings of Cambridge), mental flexibility (TMT B and ratio TMT B-A), cognitive inhibition (Stroop test), information generation (FAS fluencies, Category fluencies and Controlled oral word) and abstraction (WAIS-R Similarities) (Table 9).

**Table 9:** Executive functions: Hedge’s g effect sizes for each test, in descending order.

Test name	N studies	N CFS	N controls	Hedges'g	S.E	95% CI	*p* value	Q	*p*	I^2^ (%)	Study references
**Planning**											
Stockings of Cambridge	2	101	157	− 0.14	0.13	− 0.390 to 0.109	0.271	0.21	0.65	0.00	Capuron et al.^13^, Majer et al.^33^
**Flexibility**											
TMT B	8	255	205	0.42	0.10	0.230–0.603	0.0001	5.06	0.65	0.00	Claypoole et al.^15^, De Luca et al.^21^, Krupp et al.^30^, Lawrie et al.^31^, Michiels et al.^40^, Michiels et al.^39^, Robinson et al.^44^, Vercoulen et al.^54^
TMT B-A	2	102	73	0.10	0.16	− 0.209 to 0.403	0.536	0.26	0.61	0.00	Robinson et al.^44^, Vercoulen et al.^54^
**Inhibition**											
*Stroop*											
Interference (corrects)	2	51	61	− 0.51	0.19	− 0.885 to − 0.136	0.008	0.97	0.33	0.00	Beaumont^11^, Di Clementi et al.^57^
Interference (s) Cotation 2	2	78	40	0.40	0.20	0.004 to 0.788	0.048	0.42	0.52	0.00	Mahurin et al.^32^, Smith et al.^51^
Interference (s) Cotation 1	3	124	124	0.41	0.13	0.159 to 0.657	0.001	0.00	1.00	0.00	Cockshell et al.^17^, Cockshell et al. (2014), Ray et al.^60^
**Generation of information**											
FAS fluencies	5	180	130	− 0.37	0.12	− 0.603 to − 0.142	0.002	7.73	0.10	48.27	Cockshell et al.^17^, Joyce et al.^29^, Lawrie et al.^31^, Marcel et al.^34^, Robinson et al.^44^
Category fluencies	3	99	95	− 0.35	0.14	− 0.635 to − 0.072	0.014	2.47	0.29	19.15	Cockshell et al.^17^, Joyce et al.^29^, Marcel et al.^34^
Controlled oral word	2	42	42	− 0.02	0.22	− 0.448 to 0.406	0.922	5.77	0.02	82.68	Claypoole et al.^15^, Krupp et al.^30^
**Abstraction**											
WAIS-R similarities	2	34	33	0.04	0.24	− 0.425 to 0.510	0.86	0.06	0.80	0.00	Claypoole et al.^15^, DeLuca et al.^20^

*TMT* trail making test, *WAIS* Wechsler adult intelligence scale, *ME/CFS* myalgic encephalomyelitis/chronic fatigue syndrome, *N* number.

Statistical analysis only revealed a moderate effect size in terms of cognitive inhibition capacities (Interference [corrects responses] condition in Stroop test) (Table 9). This result therefore suggested a relative equivalence between ME/CFS and controls in terms of performance concerning executive capacities.

#### Instrumental functions

Instrumental functions include language skills (written and oral language), calculation, praxis and gnosis^84^. Ten studies evaluated the performances inherent in the instrumental functions, including six evaluating language skills through naming skills (Boston Naming Test) and vocabulary (WAIS and WAIS-R Vocabulary). Two studies proposed an evaluation of computational abilities (WAIS-R Arithmetics) and eight studies included an evaluation of visuo-constructive praxises, pencil (Copy of ROCF) or in three dimensions (WAIS-R Blocks) (Table 10).

**Table 10:** Instrumental functions: Hedge’s g effect sizes for each test, in descending order.

Test name	N studies	N CFS	N controls	Hedges'g	S.E	95% CI	*p* value	Q	*p*	I^2^ (%)	Study references
**Language**											
Boston naming test	2	59	40	0.18	0.20	− 0.219 to 0.579	0.377	0.05	0.83	0.00	Lawrie et al.^31^, Marcel et al.^34^
WAIS vocabulary	3	94	87	− 0.19	0.15	− 0.483 to 0.095	0.187	0.95	0.62	0.00	De Luca et al.^22^, Michiels et al.^40^, Short et al.^49^
WAIS-R vocabulary	2	170	101	0.08	0.13	− 0.62 to 0.331	0.501	0.31	0.58	0.00	Busichio et al.^12^, Marcel et al.^34^
**Calculation**											
Wais-R Arithmetics	2	58	53	− 0.18	0.19	− 0.550 to 0.187	0.335	0.52	0.47	0.00	Claypoole et al.^15^, De Luca et al.^22^
**Visuo-construction**											
Rey Osterreth complexe figure (copy) Cotation 2	3	92	71	− 0.39	0.16	− 0.703 to − 0.084	0.013	1.63	0.44	0.00	De Luca et al.^21,22^, Tiersky et al.^52^
Rey Osterreth complexe figure (copy) Cotation 1	3	96	98	− 0.19	0.14	− 0.470 to 0.090	0.184	3.53	0.17	43.36	Claypoole et al.^15^, Short et al.^49^, Vercoulen et al.^54^
WAIS-R blocks	3	193	113	− 0.07	0.12	− 0.305 to 0.159	0.537	0.05	0.98	0.00	Busichio et al. (2001); Claypoole et al.^15^, Lawrie et al.^31^

*WAIS* Wechsler adult intelligence scale, *ME/CFS* myalgic encephalomyelitis/chronic fatigue syndrome, *N* number.

The results of the analysis showed effect sizes below the moderate threshold and non-significant for all the observed data. This indicated that people with ME/CFS performed comparably as healthy controls for these functions (Table 10).

## Discussion

This meta-analysis highlights that ME/CFS seems to affect the cognitive sphere in a relatively heterogeneous manner with disparities between and within cognitive function. We found that ME/CFS patients have lower performances in visuo-spatial short-term memory especially concerning immediate memory. This result emanated from a comparison between two studies and de facto limits the interpretative power of the analysis. This element should therefore be considered as an evocation of possible disorders in visual immediate memory, more than an affirmation. Working memory seems to be consistent with the lack of massive executive deficit in ME/CFS patients. The verbal modality seems to be efficient. The results also suggested a slowing down affecting reading speed and graphics. The movement seems to be less clearly affected while the coordination remains quite efficient. About long-term memory, it appeared that ME/CFS patients present difficulties in several processes inherent to episodic memory (storage, retrieval, recognition) in verbal modality and with regard to recovery in visual modality.

However, these observations were not found in all the tests proposed by the included studies. This is the same observation for attention, for which the idea of a lower efficiency of the sustained attention capacities in auditory input as well as a possible weakness in visual input should be qualified by the inter-test inconsistency of these conclusions. This inconsistency can be multifactorial, in line with the heterogeneity emanating from results of previous studies^40,94^. Our methodology and inclusion criteria allowed to avoid the methodological divergences between studies concerning tests and standards used to quantify disorders^16^. Therefore, the heterogeneity of cognitive profile could lie in the variability of deficits intensity from a patient to another. In effect, ME/CFS patients may show very variable syndrome load^63–67^, even if evolving in a circumscribed framework, strictly excluding any impairment of instrumental functions. Finally, from our meta-analysis, immediate visual memory, reaction time and processing speed (reading and writing) seemed preferentially affected in ME/CFS patients, while attentional capacities and episodic memory appeared affected less constantly.

This heterogeneity also raises the question of the impact of unspecific factors as the levels of fatigue, pain and depression on cognitive performances. Regarding fatigue, its impact on the cognitive symptomatology of ME/CFS is regularly discussed and dismissed^13,94^. Interestingly, others studies dealing with multiple sclerosis came to the same conclusions^95,96^. Regarding depression, the impact of mood disturbance on cognitive abilities has been widely studied and the most affected domains are the executive functions, attention, verbal working memory and the speed of treatment^97–101^. These observations are similar with regard to pain. The pain will monopolize the attention of the patient and is the basis of ideal ruminations, which are very costly in terms of attention^102–104^. This “attentional cost" is proportional to pain intensity. Like depression, it will affect cognitive functions that rely on a good level of attention, namely executive functions and short-term memory. In fact, the higher the levels of pain and depression is, the higher the impact on cognitive symptoms is. This element could appear to generate heterogeneity between patients in terms of expression of symptoms^105^. Anyway, the neuropsychological symptomatology in ME/CFS patients cannot be reduced to the cognitive effects of depression and chronic pain. Neither chronic pain nor depression affects episodic memory^14,40,42,43^ or short-term visual memory^11,13,29,33,44^, yet significantly represented in ME/CFS patients. Altogether, this suggests that mood or pain can mediate but not cause ME/CFS symptomatology.

In the end, the clinical picture drawn by the analysis performed, despite all the measures taken to avoid bias, cannot be considered as a solid mapping of the cognitive symptoms inherent in ME/CFS. The limit in the interpretation imposed by the comparison of a number of studies sometimes restricted by cognitive domain (example of immediate visuo-spatial memory) invites to consider it more as a sketch requesting the realization of an original work to establish solidly a cognitive phenotype of ME/CFS.

On the basis of these elements and the symptomatology described through the study of all thirty-three evaluated articles, it is possible to propose a corpus of tests allowing a comprehensive evaluation of the whole cognitive sphere in ME/CFS patients. These latter, in light of our results, should benefit from a systematic evaluation to precisely delineate and quantify their cognitive impairment, considering the potential variability in pattern and intensity. The design of neuropsychological battery must allow to evaluate most finely and faithfully each function concerned, while being the least time-consuming possible to reduce bias related to fatigue. Importantly, the interpretation of results must be nuanced by the measurement of fatigue, pain and depression levels. The screening should include the evaluation of short- and long-term memory in both modalities (verbal and visual), attention (in both input channels), processing speed, executive functions and instrumental functions. Short term memory evaluation appears important in view of the symptomatology previously described and in particular in both modalities, verbal and visual^106^. It is also important to differentiate between the two components of short-term memory, namely immediate memory and working memory, in order to determine, if there is a deficit and which process is involved. Short term memory consists of the retention of an information by loop repetition system. The evaluation of immediate memory may be done by forward digit span in verbal modality (WAIS IV)^81^ and Corsi’s blocks^107^ for the visual one. The evaluation of working memory should be done by backward condition of digit span and Corsi’s blocks tests. The verbal long-term memory can be evaluated using the Free an Cued Selective Reminding Test (FCSRT)^56^. Concerning episodic memory, processes encompass three stages namely encoding, storage and retrieval^55^ to which are added the phases of recognition and consolidation of the memory trace^56^. When we suggest the presence of potential disorders affecting several of these processes, it is important to look at the question of the integrity of encoding phase^46^. Indeed, encoding is strongly influenced by the level of attention allocated to the information to be memorized. A low level of attention impacts the quality of the storage as well as the facility of recovery posteriori^108^. The choice of tests evaluating episodic memory is crucial in terms of its ability to control the quality of encoding by minimizing the impact of possible fluctuations of attention level. If so, it seems complex to distinguish between an attention or executive deficit disorder and a genuine memory deficit^108^.

In the FCSRT, the encoding mode is imposed and controlled, which avoids the potential bias of attention that may hinder the processing of the information to be memorized. The visual modality would be evaluated incidentally via the Rey-Osterrieth Complex figure^109^ (recall phase). Processing Speed would be evaluated via the TMT A and Stroop tests^110^. In these different tests, the realization times are standardized, that is to say, it is possible to compare the speed of achievement of the task of the patient to that of his/her peer group. The TMT makes it possible to take into account the speed of an act combining vision and motor gesture. As far as the Stroop is concerned, there is no motor gesture, which therefore makes it possible to obtain an estimate of the reading speed (especially in the words condition) without a potential bias of graphomotor slowing down. It appears important, as part of the understanding of neurocognitive mechanisms of ME/CFS, to assess attention in its two inputs, auditory and visual, whose processes are grouped together (sustained, divided, selective) but with different neurological pathways^111^. The dichotic listening test allows an assessment of the auditory attention^112–115^ in a relatively short period of time, making possible to avoid as much as possible a fatigue bias related to the time-consuming nature of the test situation. In this way, screening including the evaluation of mental flexibility by the TMT test, cognitive inhibition via the Stroop test and information generation via “P” and Animals fluencies have been recognized as the most sensitive for a reliable evaluation of executive functions^110^. Planning abilities would be evaluated via ROCF copy^109^. In this test, the practitioner will be particularly vigilant about the type of copy proposed by the patient, revealing, relatively accurately, his or her ability to establish an effective strategy to answer a given problem^109^. Finally, as described earlier, working memory would be evaluated via backward digit span (WAIS IV)^81^ and Corsi’s blocks^107^. Finally, the evaluation of instrumental functions would include several domains like language, ideomotors and motors praxis and visuo-construction. Language could be assessed by Boston naming test^116^ (or equivalent for other languages, as DO 80 test for French^117^, which evaluate denomination on image-based abilities. Praxis could be assessed by dedicated battery such as Mahieux’ praxies battery for French patients (Praxies)^118^ and ROCF (visuo-constructive praxies)^109^ which have the particularity of being sensitive and not very time consuming. To control the fatigue, mood and pain biases, self-evaluation scales could be proposed at the beginning of the assessment.

Cognitive troubles have a marked impact on the disability and the quality of life of ME/CFS patients. An optimum neuropsychological evaluation should fulfill the following requirements: (1) to be standardized and homogeneous across different centers taking care of ME/CFS patients; (2) to make possible delineating deficits and discriminating functions never altered through a most complete and detailed screening; (3) to make possible a stratification of patients according to their neuropsychological profiles; and (4) to be short enough to avoid fatigue biases. Henri Mondor group proposed an operative battery adequately shaped for ME/CFS, which does not exceed duration of 75 min making it endurable for a majority of patients and therefore fully suitable for the evaluation of ME/CFS patients (Table 11). This battery has already proven its applicability for the neuropsychological evaluation of patients complaining from cognitive difficulties in the context of routine care^119^.

**Table 11:** Henri Mondor battery for the neuropsychological assessment of patients with chronic fatigue and cognitive complaints.

**Pain, fatigue, depression**	**Memories**
BDI II (depression)	Forward digit span
VAS (Pain)	FCSRT
VAS (fatigue)	Rey figure recall (3 min)
**Executive functions and attention**	**Instrumental functions**
Backward digit span	Praxies
FCSRT	Boston naming test or DO 80 (French speaking patients)
TMT A & B	
Stroop (GREFEX version)	**Dichotic listening**
« P » and « Animal » fluencies	Word and sentences conditions
Rey figure (copy)	
Zazzo’s cancellation test	

*BDI* beck depression inventory, *VAS* visual analogic scale, *FCSRT* free and cued recall selective reminding test, *TMT* trail making test, *DO* Dénomination Orale (Deloche & Hannequin, 1997).

The idea of a complete screening is to tend towards the identification of potentially typical cognitive disorders which can embody diagnostic call points. This would, in the long term, establish a limited duration assessment of allowing minimizing the fatigue bias particularly inherent in the pathology. In sum, the determination of the presence of cognitive disorders in the ME/CFS seems closely related to the modalities of neuropsychological assessment. Therefore, it seems sound to consider that the determination of a robust cognitive picture of ME/CFS will require a comprehensive neuropsychological battery integrating the intrinsic complexity of the pathology.

This meta-analysis, based on compliance with rigorous methodological criteria, leads to the idea of a common cognitive phenotype in patients with ME/CFS. Nevertheless, we highlighted limits regarding the heterogeneity between some studies, in particular concerning reaction time or, to a lesser extent, visual short-term memory. Another limitation is the small number of studies for some cognitive processes like visual short-term memory. Although these limitations do not invalidate the main findings, the idea of a common cognitive profile in patients with ME/CFS needs further research to have a strong specific profile in the future.

For these reasons, original studies dealing with neuropsychological profile of ME/CFS are crucially needed. Our literature review and meta-analysis supports the view of the heterogeneity of cognitive profile in ME/CFS. It also makes it possible to deduce the potential mechanism through the description of a well-defined syndrome framework integrating disorders with variable expression and the constant preservation of instrumental functions. In the context of clinical neuropsychology research in the field of ME/CFS, it is needed for protocols to include a comprehensive evaluation of cognitive functions, and the choice of sensitive tests avoiding as much as possible testing bias and whose number for each function group evaluated must be consistent with their respective complexity (Table 12). It also appears, given the heterogeneity in the expression of cognitive disorders, to work on larger cohorts in order to categorize with precision the different cognitive impairment profiles emerging from this diverse symptomatology. With this in mind, it also appears important to integrate an assessment of the patient levels of depression, fatigue and pain.

**Table 12:** Take home message.

1	Myalgic encephalomyelitis/chronic fatigue syndrome (ME/CFS) commonly associates with cognitive complaints
2	Neuropsychological testing is required for appropriately descripting and quantifying the cognitive impairment in ME/CFS patients
3	ME/CFS-associated cognitive impairment typically affects visuo-spatial immediate memory, reaction time, reading speed and the speed of the graphic gesture;
4	Episodic verbal and visual memories and attentional abilities may be also impaired
5	Instrumental functions appear constantly preserved

Future research also need to include brain imaging to assess the hypothesis of a possible neurological dysfunction at the origin of the cognitive disorders in ME/CFS. Indeed, visuospatial processing and memory are thought to be supported by the fronto-temporo-parietal and occipital areas^120–122^. This could therefore be compared to imaging data reflecting hypometabolism concerning the posterior associative areas^123,124^. The mechanisms underlying cognitive dysfunction in ME/CFS are unclear. Neuroinflammation can be visualized through PET brain imaging using a radioligand for translocator protein (TSPO), a protein belonging to mitochondrial permeability transition pore (MPTP) complex and produced when microglia become activated^125^. This approach showed a correlation between cognitive impairment in ME/CFS patients and PET-signal, especially in a region between mid-pons and thalamus^126^, so supporting the hypothesis of neuroinflammation in ME/CFS.

Finally, in order to obtain a solid estimate of the cognitive symptomatology, future research should systematically report psychotropic drugs treatment^127^ in the process of patient inclusion and in the treatment of data, in order to avoid bias in data interpretation. The categorization of various forms of dysfunction will allow in the future offering cognitive remediation support adapted to each problem. It will also be important to consider other symptoms such as fatigue, pain and morbid interactions between them. Indeed, multi-domain disorders (fatigue, pain, cognition) have an impact on the professional, family and social sphere of patients. This induces significant effects on the mood level. This phenomenon is accentuated by the difficulties in recognizing the arduousness of the experience in this context of multiple but invisible disability. The future objective could be to study the representation of the pathology among all the members constituting the environment of the patients (professionals, caregivers) and in patients themselves. This is to quantify the impact cognitive disorders on the patient's representation of his pathology, but also on his mood. Could also be approached the impact of issues of representation on the thymic sphere and the impact of mood on the feeling of fatigue and pain. Chronic fatigue syndrome is a multiple entry entity^68^, with multiple symptomatology and patients must be the subject of a clinical reading at the height of the complexity of the pathology.

## Supplementary Information

Supplementary Information 1.

Supplementary Information 2.
